# Supplementary material for: WT1 Pulsed Human CD141+ Dendritic Cell Vaccine Has High Potential in Solid Tumor-Targeted Immunotherapy
Source: Int J Mol Sci. 2023 Jan 12;24(2):1501. doi: 10.3390/ijms24021501 (PMC9864659; doi:10.3390/ijms24021501)
Supplement: Supplementary file 1 [file ijms-24-01501-s001.zip › ijms-2025327-supplementary.pdf]

Table S1-1. Summary of Clinical Signs (Dosing period)

Sex: Male

Group / Dose (cells/animal)	No. of animals	Clinical sign	No. of animals affected	Days 1 ~ 42 - observed during the days
G1<br>0	15	NOA	15	Days 1 ~ 42
G2<br>Saline: 0	15	NOA	15	Days 1 ~ 42
G3<br>3.4×10^4	10	NOA	10	Days 1 ~ 42
G4<br>1.7×10^5	15	NOA	15	Days 1 ~ 42

Sex: Female

Group / Dose (cells/animal)	No. of animals	Clinical sign	No. of animals affected	Days 1 ~ 42 - observed during the days
G1<br>0	15	NOA	15	Days 1 ~ 42
G2<br>Saline: 0	15	NOA	15	Days 1 ~ 42
G3<br>3.4×10^4	10	NOA	10	Days 1 ~ 42
G4<br>1.7×10^5	15	NOA	15	Days 1 ~ 42

NOA: No Observable Abnormality

Table S1-2. Summary of Clinical Signs (Recovery period)

Sex: Male

Group / Dose (cells/animal)	No. of animals	Clinical sign	No. of animals affected	Days 43 ~ 56 - observed during the days
G1<br>0	5	NOA	5	Days 43 ~ 56
G2<br>Saline: 0	5	NOA	5	Days 43 ~ 56
G4<br>1.7×10^{5}	5	NOA	5	Days 43 ~ 56

Sex: Female

Group / Dose (cells/animal)	No. of animals	Clinical sign	No. of animals affected	Days 43 ~ 56 - observed during the days
G1<br>0	5	NOA	5	Days 43 ~ 56
G2<br>Saline: 0	5	NOA	5	Days 43 ~ 56
G4<br>1.7×10^{5}	5	NOA	5	Days 43 ~ 56

NOA: No Observable Abnormality

Table S2. Mean Body Weights

Sex: Male (g)

Group / Dose (cells/animal)		Week									
		Dosing period							Recovery period		
		0	1	2	3	4	5	6	6	7	8
G1	Mean	19.8	21.5	22.7	23.7	24.5	25.2	25.7	26.0	26.3	26.5
0	S.D.	0.9	0.9	1.0	1.0	1.2	1.1	1.1	1.4	1.4	1.2
	N	15	15	15	15	15	15	15	5	5	5
G2	Mean	19.9	21.5	22.7	23.9	24.7	25.4	26.0	26.6	27.4	28.0
Saline: 0	S.D.	0.8	0.8	1.0	1.2	1.3	1.2	1.3	1.3	1.8	1.5
	N	15	15	15	15	15	15	15	5	5	5
G3	Mean	19.9	21.6	22.7	23.3	24.2	24.9	25.4			
3.4×10^4	S.D.	0.9	1.2	1.5	1.3	1.0	1.0	1.1			
	N	10	10	10	10	10	10	10			
G4	Mean	19.8	21.4	22.7	24.2	25.0	25.6	26.1	26.0	26.2	26.4
1.7×10^5	S.D.	1.0	1.0	1.0	1.1	1.3	1.6	1.6	1.5	1.1	1.3
	N	15	15	15	15	15	15	15	5	5	5

Sex: Female (g)

Group / Dose (cells/animal)		Week									
		Dosing period							Recovery period		
		0	1	2	3	4	5	6	6	7	8
G1	Mean	17.6	18.4	19.9	20.6	21.7	22.4	22.8	22.4	22.4	22.9
0	S.D.	0.8	0.8	1.8	1.1	1.1	1.1	1.1	0.8	0.8	0.6
	N	15	15	15	15	15	15	15	5	5	5
G2	Mean	17.5	18.6	19.6	20.7	22.1	22.6	23.2	22.2	23.3	23.1
Saline: 0	S.D.	0.9	0.9	0.9	1.2	1.2	0.7	1.0	0.5	0.3	1.0
	N	15	15	15	15	15	15	15	5	5	5
G3	Mean	17.5	18.7	19.7	21.0	22.2	22.5	23.0			
3.4×10^4	S.D.	0.5	1.0	0.8	0.9	0.9	1.4	1.0			
	N	10	10	10	10	10	10	10			
G4	Mean	17.5	18.4	19.3	20.9	22.1	22.4	23.1	23.7	23.3	24.1
1.7×10^5	S.D.	0.7	1.2	1.2	1.3	1.4	1.8	1.3	1.1	0.7	0.6
	N	15	15	15	15	15	15	15	5	5	5

Table S3. Mean Food Consumption

Sex: Male (g/day)

Group / Dose (cells/animal)		Week								
		Dosing period							Recovery period	
		0	1	2	3	4	5	6	7	8
G1	Mean	7.1	4.8	4.8	5.0	5.3	6.2	6.0	5.6	5.4
0	S.D.	2.4	0.7	0.6	0.6	1.1	1.9	2.1	1.3	0.9
	N	15	15	15	15	15	15	15	5	5
G2	Mean	6.9	4.5	4.7	4.8	5.1	5.7	5.9	7.0	6.6
Saline: 0	S.D.	1.2	0.3	0.2	0.5	0.7	0.7	1.7	2.4	1.0
	N	15	15	15	15	15	15	15	5	5
G3	Mean	6.3	4.5	4.6	4.7	4.6	5.3	5.0		
3.4×10^4	S.D.	0.9	0.3	0.4	0.6	0.4	0.5	0.6		
	N	10	10	10	10	10	10	10		
G4	Mean	6.0	4.7	4.7	4.9	4.9	5.3	5.1	5.5	5.2
1.7×10^5	S.D.	0.8	0.4	0.6	0.5	0.5	0.6	0.9	1.4	0.7
	N	15	15	15	15	15	15	15	5	5

Sex: Female (g/day)

Group / Dose (cells/animal)		Week								
		Dosing period							Recovery period	
		0	1	2	3	4	5	6	7	8
G1	Mean	5.7	4.3	4.3	4.5	4.6	4.9	4.9	4.7	5.0
0	S.D.	0.9	0.3	0.3	0.4	0.6	0.5	0.6	0.9	0.7
	N	15	15	15	15	15	15	15	5	5
G2	Mean	5.6	4.5	4.3	4.4	4.6	4.9	5.0	5.2	5.1
Saline: 0	S.D.	1.1	0.3	0.5	0.6	0.5	0.3	0.4	0.3	0.6
	N	15	15	15	15	15	15	15	5	5
G3	Mean	5.5	4.4	4.3	4.4	4.7	4.8	4.8		
3.4×10^4	S.D.	1.0	0.4	0.3	0.4	0.3	0.7	0.4		
	N	10	10	10	10	10	10	10		
G4	Mean	5.3	4.3	4.2	4.3	4.5	4.7	4.9	4.9	5.0
1.7×10^5	S.D.	0.7	0.4	0.3	0.4	0.4	0.6	0.5	0.1	0.2
	N	15	15	15	15	15	15	15	5	5

Table S4-1. Summary of Ophthalmological Examination (Main group)

Sex: Male

Group / Dose (cells/animal)	No. of animals	Findings	Right eye				Left eye			
			Pupil light reflex	Anterior segment	Trans-parent media	Fundus	Pupil light reflex	Anterior segment	Trans-parent media	Fundus
G1<br>0	10	Normal	10	10	10	10	10	10	10	10
G2<br>Saline: 0	10	Normal	10	10	10	10	10	10	10	10
G3<br>3.4×10^4	10	Normal	10	10	10	10	10	10	10	10
G4<br>1.7×10^5	10	Normal	10	10	10	10	10	10	10	10

Sex: Female

Group / Dose (cells/animal)	No. of animals	Findings	Right eye				Left eye			
			Pupil light reflex	Anterior segment	Trans-parent media	Fundus	Pupil light reflex	Anterior segment	Trans-parent media	Fundus
G1<br>0	10	Normal<br>Opacity	10	10	10	10	9<br>1	10	9<br>1	9
G2<br>Saline: 0	10	Normal	10	10	10	10	10	10	10	10
G3<br>3.4×10^4	10	Normal	10	10	10	10	10	10	10	10
G4<br>1.7×10^5	10	Normal	10	10	10	10	10	10	10	10

Table S4-2. Summary of Ophthalmological Examination (Recovery group)

Sex: Male

Group / Dose (cells/animal)	No. of animals	Findings	Right eye				Left eye			
			Pupil light reflex	Anterior segment	Trans-parent media	Fundus	Pupil light reflex	Anterior segment	Trans-parent media	Fundus
G1<br>0	5	Normal	5	5	5	5	5	5	5	5
G2<br>Saline: 0	5	Normal	5	5	5	5	5	5	5	5
G4<br>1.7×10^5	5	Normal	5	5	5	5	5	5	5	5

Sex: Female

Group / Dose (cells/animal)	No. of animals	Findings	Right eye				Left eye			
			Pupil light reflex	Anterior segment	Trans-parent media	Fundus	Pupil light reflex	Anterior segment	Trans-parent media	Fundus
G1<br>0	5	Normal	5	5	5	5	5	5	5	5
G2<br>Saline: 0	5	Normal	5	5	5	5	5	5	5	5
G4<br>1.7×10^5	5	Normal	5	5	5	5	5	5	5	5

Table S5-1. Summary of Urinalysis Results (Main group)

Sex		Male				Female			
Group /		G1	G2	G3	G4	G1	G2	G3	G4
Dose (cells/animal)		0	Saline: 0	3.4×10^4	1.7×10^5	0	Saline: 0	3.4×10^4	1.7×10^5
No. of animals		10	10	10	10	10	10	10	10
pH	5		1	1		1	2	3	5
	6	5	1	2	2	3	2	4	1
	7	3	4	5	5	6	5	1	3
	8	2	2	1	2		1	2	1
	9		2	1	1				
Protein	Neg.	10	10	10	10	10	10	10	10
(mg/dL)	1+								
	2+								
	3+								
Glucose	Normal	10	10	10	10	10	10	10	10
(mg/dL)	1+								
	2+								
	3+								
	4+								
Ketone body	Neg.	10	9	9	8	8	9	7	7
(mg/dL)	1+		1	1	2	2	1	3	3
	2+								
	3+								
Bilirubin	Neg.	10	10	10	10	10	10	9	10
(mg/dL)	1+							1	
	2+								
	3+								
Occult blood	Neg.	9	10	10	10	10	10	10	10
(Ery/μL)	1+	1							
	2+								
	3+								
	4+								

Table S5-2. Summary of Urinalysis Results (Recovery group)

Sex		Male			Female		
Group /		G1	G2	G4	G1	G2	G4
Dose (cells/animal)		0	Saline: 0	1.7×10^5	0	Saline: 0	1.7×10^5
No. of animals		5	5	5	5	5	5
pH	5			1	1	1	1
	6	2	1	1	1		1
	7	3	2	2	3	4	2
	8		1	1			1
	9		1				
Protein	Neg.	5	4	4	3	5	5
(mg/dL)	1+		1	1	2		
	2+						
	3+						
Glucose	Normal	5	5	5	5	5	5
(mg/dL)	1+						
	2+						
	3+						
	4+						
Ketone body	Neg.	4	4	4	3	1	5
(mg/dL)	1+	1	1	1	2	4	
	2+						
	3+						
Bilirubin	Neg.	5	5	5	5	5	5
(mg/dL)	1+						
	2+						
	3+						
Occult blood	Neg.	5	5	5	4	5	5
(Ery/µL)	1+				1		
	2+						
	3+						
	4+						

Table S6-1. Mean Hematological Parameters (Main group)

Sex: Male

Group / Dose (cells/animal)		RBC ($\times10^6$ cells/μL)	HGB (g/dL)	HCT (%)	RBC Indices			PLT ($\times10^3$ cells/μL)
					MCV (fL)	MCH (pg)	MCHC (g/dL)	
G1	Mean	10.37	15.1	47.1	45.5	14.6	32.1	1370
0	S.D.	0.44	0.7	1.8	0.7	0.1	0.4	232
	N	10	10	10	10	10	10	10
G2	Mean	10.59	15.4	48.1	45.4	14.5	32.0	1196
Saline: 0	S.D.	0.33	0.5	1.4	0.5	0.2	0.5	323
	N	10	10	10	10	10	10	10
G3	Mean	10.38	15.0	46.8	45.1	14.5	32.1	1233
3.4×10^4	S.D.	0.42	0.6	1.7	0.4	0.2	0.4	238
	N	10	10	10	10	10	10	10
G4	Mean	10.38	15.1	47.3	45.5	14.6	32.0	1356
1.7×10^5	S.D.	0.29	0.4	1.2	0.6	0.1	0.3	209
	N	10	10	10	10	10	10	10

Group / Dose (cells/animal)		WBC ($\times10^3$ cells/μL)	WBC Differential Counting (%)					Reti (%)
			NEU	LYM	MONO	EOS	BASO	
G1	Mean	2.43	10.5	86.8	2.4	0.3	0.0	3.41
0	S.D.	0.79	5.4	5.1	0.6	0.4	0.0	0.30
	N	10	10	10	10	10	10	10
G2	Mean	2.82	8.8	88.3	2.4	0.5	0.0	3.56
Saline: 0	S.D.	1.61	2.6	3.0	0.7	0.6	0.0	0.56
	N	10	10	10	10	10	10	10
G3	Mean	3.38	7.2	88.9	2.6	1.4	0.0	3.49
3.4×10^4	S.D.	0.88	1.7	2.7	0.8	1.2	0.0	0.34
	N	10	10	10	10	10	10	10
G4	Mean	3.12	7.9	89.1	2.4	0.7	0.0	3.37
1.7×10^5	S.D.	2.09	2.7	3.2	0.8	1.0	0.0	0.20
	N	10	10	10	10	10	10	10

Table S6-1. (Continued)

Sex: Female

Group / Dose (cells/animal)		RBC ($\times10^6$ cells/μL)	HGB (g/dL)	HCT (%)	RBC Indices			PLT ($\times10^3$ cells/μL)
					MCV (fL)	MCH (pg)	MCHC (g/dL)	
G1	Mean	9.80	14.6	45.8	46.8	14.9	31.8	1229
0	S.D.	0.35	0.4	1.1	0.7	0.2	0.4	97
	N	10	10	10	10	10	10	10
G2	Mean	9.68	14.5	45.3	46.8	15.0	32.0	1211
Saline: 0	S.D.	0.36	0.4	1.4	0.6	0.2	0.2	135
	N	10	10	10	10	10	10	10
G3	Mean	9.63	14.4	45.1	46.8	15.0	32.0	1161
3.4×10^4	S.D.	0.18	0.4	0.7	0.4	0.2	0.4	174
	N	10	10	10	10	10	10	10
G4	Mean	9.61	14.3	44.8	46.6	14.8	31.9	1281
1.7×10^5	S.D.	0.28	0.3	1.1	0.4	0.1	0.3	70
	N	10	10	10	10	10	10	10

Group / Dose (cells/animal)		WBC ($\times10^3$ cells/μL)	WBC Differential Counting (%)					Reti (%)
			NEU	LYM	MONO	EOS	BASO	
G1	Mean	6.35	6.2	85.5	4.0	4.2	0.1	3.64
0	S.D.	2.43	1.6	1.4	0.6	1.5	0.1	0.52
	N	10	10	10	10	10	10	10
G2	Mean	6.14	6.6	87.6 *	3.7	2.1 **	0.1	3.33
Saline: 0	S.D.	2.80	1.6	1.3	0.9	1.0	0.1	0.51
	N	10	10	10	10	10	10	10
G3	Mean	6.55	7.1	84.7	4.2	3.9	0.1	3.86
3.4×10^4	S.D.	2.79	2.0	1.6	0.7	1.1	0.1	0.87
	N	10	10	10	10	10	10	10
G4	Mean	5.63	6.8	84.9	4.0	4.2	0.1	3.59
1.7×10^5	S.D.	1.66	1.6	2.6	0.8	1.0	0.1	0.60
	N	10	10	10	10	10	10	10

Significantly different from control by Dunnett's t-test: * $p<0.05$, ** $p<0.01$.

Table S6-2. Mean Hematological Parameters (Recovery group)

Sex: Male

Group / Dose (cells/animal)		RBC ($\times 10^6$ cells/μL)	HGB (g/dL)	HCT (%)	RBC Indices			PLT ($\times 10^3$ cells/μL)
					MCV (fL)	MCH (pg)	MCHC (g/dL)	
G1	Mean	10.37	14.8	46.7	45.1	14.3	31.7	1396
0	S.D.	0.27	0.4	1.0	0.4	0.1	0.4	274
	N	5	5	5	5	5	5	5
G2	Mean	10.17	14.5	46.2	45.4	14.3	31.3	1324
Saline: 0	S.D.	0.20	0.2	0.6	0.4	0.1	0.2	258
	N	5	5	5	5	5	5	5
G4	Mean	10.02	14.4	45.5	45.4	14.3	31.5	1392
1.7×10^5	S.D.	0.19	0.3	0.8	0.4	0.2	0.3	225
	N	5	5	5	5	5	5	5

Group / Dose (cells/animal)		WBC (K/μL)	WBC Differential Counting (%)					Reti (%)
			NEU	LYM	MONO	EOS	BASO	
G1	Mean	9.58	5.4	88.2	3.7	2.6	0.1	3.38
0	S.D.	2.23	1.0	1.2	0.6	0.2	0.0	0.33
	N	5	5	5	5	5	5	5
G2	Mean	7.49	5.0	88.8	3.9	2.2	0.1	3.24
Saline: 0	S.D.	1.58	0.9	1.8	1.0	0.4	0.1	0.14
	N	5	5	5	5	5	5	5
G4	Mean	9.15	5.1	88.4	4.0	2.4	0.1	3.73
1.7×10^5	S.D.	1.52	1.1	2.0	0.7	0.9	0.0	0.26
	N	5	5	5	5	5	5	5

Table S6-2. (Continued)

Sex: Female

Group / Dose (cells/animal)		RBC (M/μL)	HGB (g/dL)	HCT (%)	RBC Indices MCV (fL)	RBC Indices MCH (pg)	RBC Indices MCHC (g/dL)	PLT (K/μL)
G1	Mean	9.84	14.3	45.2	45.9	14.6	31.7	1283
0	S.D.	0.23	0.5	1.2	0.7	0.2	0.4	223
	N	5	5	5	5	5	5	5
G2	Mean	10.08	14.7	46.4	46.0	14.6	31.8	1292
Saline: 0	S.D.	0.24	0.4	1.3	0.4	0.1	0.2	74
	N	5	5	5	5	5	5	5
G4	Mean	9.87	14.4	45.3	45.9	14.6	31.8	1338
1.7×10^5	S.D.	0.39	0.4	1.3	0.5	0.3	0.3	62
	N	5	5	5	5	5	5	5

Group / Dose (cells/animal)		WBC (K/μL)	WBC Differential Counting (%) NEU	LYM	MONO	EOS	BASO	Reti (%)
G1	Mean	4.74	7.2	86.5	3.8	2.5	0.0	3.42
0	S.D.	1.69	1.6	1.1	1.0	0.6	0.0	0.79
	N	5	5	5	5	5	5	5
G2	Mean	4.88	7.0	87.4	3.4	2.0	0.1	3.48
Saline: 0	S.D.	1.45	1.1	1.4	0.6	0.5	0.1	0.44
	N	5	5	5	5	5	5	5
G4	Mean	4.26	6.2	86.4	3.9	3.4	0.0	3.16
1.7×10^5	S.D.	1.02	1.0	1.8	1.6	1.1	0.1	0.20
	N	5	5	5	5	5	5	5

Table S7-1. Mean Clinical Chemistry (Main group)

Sex: Male

Group / Dose (cells/animal)		ALT (U/L)	AST (U/L)	ALP (U/L)	Glu (mg/dL)	BUN (mg/dL)	Crea (mg/dL)	T-Bili (mg/dL)
G1	Mean	35.5	83.6	279.0	250	52.7	0.37	0.03
0	S.D.	7.2	29.1	32.1	36	15.5	0.04	0.02
	N	10	10	10	10	10	10	10
G2	Mean	41.8	84.3	293.1	262	43.0	0.38	0.04
Saline: 0	S.D.	15.9	31.6	20.1	48	12.7	0.05	0.04
	N	10	10	10	10	10	10	10
G3	Mean	40.9	79.8	268.8	269	41.7	0.39	0.04
3.4×10^4	S.D.	14.2	34.6	27.6	21	13.5	0.03	0.02
	N	10	10	10	10	10	10	10
G4	Mean	37.7	79.1	264.4	261	47.0	0.38	0.03
1.7×10^5	S.D.	6.3	29.6	21.6	22	11.6	0.02	0.02
	N	10	10	10	10	10	10	10

Group / Dose (cells/animal)		T-Chol (mg/dL)	TG (mg/dL)	TP (g/dL)	Alb (g/dL)	A/G ratio	P (mg/dL)	Ca (mg/dL)
G1	Mean	108	45	5.1	1.8	0.56	8.68	9.5
0	S.D.	11	14	0.3	0.1	0.02	0.84	0.4
	N	10	10	10	10	10	10	10
G2	Mean	103	47	5.1	1.8	0.55	8.44	9.6
Saline: 0	S.D.	19	15	0.2	0.1	0.03	1.09	0.2
	N	10	10	10	10	10	10	10
G3	Mean	105	50	5.2	1.8	0.54	7.48 *	9.6
3.4×10^4	S.D.	12	16	0.3	0.1	0.03	0.85	0.3
	N	10	10	10	10	10	10	10
G4	Mean	103	43	5.2	1.8	0.54	8.33	9.6
1.7×10^5	S.D.	11	8	0.3	0.1	0.02	0.71	0.2
	N	10	10	10	10	10	10	10

Significantly different from control by Dunnett's t-test: * $p<0.05$.

Table S7-1. (Continued)

Sex: Female

Group / Dose (cells/animal)		ALT (U/L)	AST (U/L)	ALP (U/L)	Glu (mg/dL)	BUN (mg/dL)	Crea (mg/dL)	T-Bili (mg/dL)
G1	Mean	28.6	77.0	379.0	246	21.2	0.32	0.05
0	S.D.	7.6	26.3	22.4	32	7.3	0.03	0.03
	N	10	10	10	10	10	10	10
G2	Mean	29.9	75.5	391.1	258	26.2	0.34	0.07
Saline: 0	S.D.	10.3	28.0	34.3	31	7.4	0.03	0.05
	N	10	10	10	10	10	10	10
G3	Mean	28.5	66.8	378.5	243	20.9	0.33	0.08
3.4×10^4	S.D.	5.6	14.2	54.3	38	4.1	0.03	0.04
	N	10	10	10	10	10	10	10
G4	Mean	28.0	74.3	368.8	257	24.5	0.33	0.07
1.7×10^5	S.D.	4.4	17.1	43.6	34	4.3	0.04	0.02
	N	10	10	10	10	10	10	10

Group / Dose (cells/animal)		T-Chol (mg/dL)	TG (mg/dL)	TP (g/dL)	Alb (g/dL)	A/G ratio	P (mg/dL)	Ca (mg/dL)
G1	Mean	89	33	4.7	1.7	0.56	7.70	9.0
0	S.D.	5	7	0.2	0.1	0.03	0.72	0.3
	N	10	10	10	10	10	10	10
G2	Mean	89	34	4.6	1.7	0.58	7.56	9.0
Saline: 0	S.D.	11	8	0.3	0.1	0.02	1.04	0.3
	N	10	10	10	10	10	10	10
G3	Mean	85	29	4.9	1.7	0.54	7.70	9.1
3.4×10^4	S.D.	11	5	0.3	0.1	0.03	0.43	0.3
	N	10	10	10	10	10	10	10
G4	Mean	89	35	4.8	1.7	0.55	7.67	8.9
1.7×10^5	S.D.	7	7	0.2	0.0	0.02	0.80	0.1
	N	10	10	10	10	10	10	10

Table S7-2. Mean Clinical Chemistry (Recovery group)

Sex: Male

Group / Dose (cells/animal)		ALT (U/L)	AST (U/L)	ALP (U/L)	Glu (mg/dL)	BUN (mg/dL)	Crea (mg/dL)	T-Bili (mg/dL)
G1	Mean	36.1	141.3	229.1	261	29.2	0.42	0.03
0	S.D.	6.0	46.9	23.2	21	3.4	0.03	0.02
	N	5	5	5	5	5	5	5
G2	Mean	31.1	110.9	212.0	277	33.2	0.43	0.02
Saline: 0	S.D.	3.9	39.9	18.1	19	6.4	0.03	0.01
	N	5	5	5	5	5	5	5
G4	Mean	31.9	120.4	225.7	251	31.9	0.41	0.06
$1.7×10^5$	S.D.	4.1	38.7	14.1	32	9.8	0.03	0.06
	N	5	5	5	5	5	5	5

Group / Dose (cells/animal)		T-Chol (mg/dL)	TG (mg/dL)	TP (g/dL)	Alb (g/dL)	A/G ratio	P (mg/dL)	Ca (mg/dL)
G1	Mean	101	39	4.6	1.6	0.53	6.54	9.2
0	S.D.	3	3	0.0	0.0	0.00	0.98	0.2
	N	5	5	5	5	5	5	5
G2	Mean	99	41	4.6	1.6	0.53	6.72	9.1
Saline: 0	S.D.	5	3	0.1	0.0	0.03	0.56	0.3
	N	5	5	5	5	5	5	5
G4	Mean	86 #	34	4.6	1.6	0.53	7.42	9.1
$1.7×10^5$	S.D.	11	11	0.2	0.1	0.01	1.24	0.2
	N	5	5	5	5	5	5	5

Significantly different from control by Steel test: # $p<0.05$.

Table S7-2. (Continued)

Sex: Female

Group / Dose (cells/animal)		ALT (U/L)	AST (U/L)	ALP (U/L)	Glu (mg/dL)	BUN (mg/dL)	Crea (mg/dL)	T-Bili (mg/dL)
G1	Mean	35.5	141.4	331.4	242	31.8	0.37	0.03
0	S.D.	5.3	30.5	52.6	27	5.7	0.04	0.02
	N	5	5	5	5	5	5	5
G2	Mean	36.5	156.9	351.0	265	24.9	0.37	0.03
Saline: 0	S.D.	5.5	29.4	18.1	29	3.5	0.03	0.02
	N	5	5	5	5	5	5	5
G4	Mean	41.0	140.4	326.9	256	32.4	0.38	0.02
1.7×10^5	S.D.	13.9	34.3	24.4	37	8.0	0.03	0.00
	N	5	5	5	5	5	5	5

Group / Dose (cells/animal)		T-Chol (mg/dL)	TG (mg/dL)	TP (g/dL)	Alb (g/dL)	A/G ratio	P (mg/dL)	Ca (mg/dL)
G1	Mean	86	33	4.7	1.7	0.55	8.84	9.0
0	S.D.	5	8	0.2	0.1	0.00	0.87	0.1
	N	5	5	5	5	5	5	5
G2	Mean	90	33	4.8	1.7	0.57	8.95	9.0
Saline: 0	S.D.	10	6	0.2	0.1	0.03	0.72	0.2
	N	5	5	5	5	5	5	5
G4	Mean	87	30	4.8	1.7	0.54	8.29	9.0
1.7×10^5	S.D.	2	4	0.2	0.1	0.03	0.23	0.3
	N	5	5	5	5	5	5	5

Table S8-1. Mean Absolute Organ Weights (Main group)

Sex: Male (g)

Group / Dose (cells/animal)		B.W.	Brain	Pituitary	Heart	Lung	Liver
G1	Mean	24.3	0.424	0.0015	0.125	0.138	1.267
0	S.D.	1.2	0.012	0.0003	0.007	0.006	0.082
	N	10	10	10	10	10	10
G2	Mean	24.5	0.430	0.0016	0.127	0.141	1.257
Saline: 0	S.D.	1.2	0.018	0.0005	0.012	0.014	0.156
	N	10	10	10	10	10	10
G3	Mean	24.6	0.426	0.0015	0.123	0.151	1.358
3.4×10^4	S.D.	1.4	0.012	0.0002	0.012	0.022	0.128
	N	10	10	10	10	10	10
G4	Mean	24.8	0.428	0.0016	0.131	0.143	1.320
1.7×10^5	S.D.	1.8	0.014	0.0003	0.012	0.009	0.120
	N	10	10	10	10	10	10

Group / Dose (cells/animal)		Spleen	Kidney	Adrenal gland	Testis	Prostate
G1	Mean	0.057	0.316	0.0041	0.176	0.0088
0	S.D.	0.004	0.019	0.0008	0.021	0.0045
	N	10	10	10	10	10
G2	Mean	0.064	0.333	0.0041	0.189	0.0081
Saline: 0	S.D.	0.015	0.039	0.0013	0.027	0.0032
	N	10	10	10	10	10
G3	Mean	0.064	0.319	0.0039	0.165	0.0099
3.4×10^4	S.D.	0.009	0.023	0.0010	0.018	0.0027
	N	10	10	10	10	10
G4	Mean	0.065	0.335	0.0044	0.188	0.0086
1.7×10^5	S.D.	0.009	0.025	0.0011	0.020	0.0028
	N	10	10	10	10	10

Table S8-1. (Continued)

Sex: Female								(g)
Group / Dose (cells/animal)		B.W.	Brain	Pituitary	Heart	Lung	Liver	
G1	Mean	22.2	0.453	0.0020	0.114	0.155	1.168	
0	S.D.	0.9	0.018	0.0003	0.010	0.016	0.105	
	N	10	10	10	10	10	10	
G2	Mean	22.9	0.457	0.0020	0.117	0.158	1.193	
Saline: 0	S.D.	1.3	0.017	0.0003	0.011	0.014	0.125	
	N	10	10	10	10	10	10	
G3	Mean	22.3	0.457	0.0022	0.118	0.156	1.141	
3.4×10^4	S.D.	1.0	0.017	0.0007	0.012	0.010	0.119	
	N	10	10	10	10	10	10	
G4	Mean	22.2	0.457	0.0018	0.115	0.159	1.172	
1.7×10^5	S.D.	1.1	0.010	0.0004	0.004	0.019	0.075	
	N	10	10	10	10	10	10	

Group / Dose (cells/animal)		Spleen	Kidney	Adrenal gland	Ovary	Uterus
G1	Mean	0.087	0.275	0.0082	0.0076	0.116
0	S.D.	0.009	0.025	0.0012	0.0013	0.037
	N	10	10	10	10	10
G2	Mean	0.084	0.272	0.0068	0.0062	0.105
Saline: 0	S.D.	0.012	0.024	0.0016	0.0008	0.041
	N	10	10	10	10	10
G3	Mean	0.095	0.269	0.0071	0.0070	0.114
3.4×10^4	S.D.	0.012	0.013	0.0014	0.0015	0.049
	N	10	10	10	10	10
G4	Mean	0.088	0.265	0.0074	0.0067	0.110
1.7×10^5	S.D.	0.013	0.022	0.0016	0.0023	0.036
	N	10	10	10	10	10

Table S8-2. Mean Absolute Organ Weights (Recovery group)

Sex: Male (g)

Group / Dose (cells/animal)		B.W.	Brain	Pituitary	Heart	Lung	Liver
G1	Mean	25.9	0.423	0.0014	0.125	0.138	1.329
0	S.D.	1.3	0.004	0.0002	0.006	0.003	0.069
	N	5	5	5	5	5	5
G2	Mean	27.0	0.421	0.0014	0.129	0.145	1.405
Saline: 0	S.D.	1.3	0.014	0.0002	0.010	0.013	0.073
	N	5	5	5	5	5	5
G4	Mean	25.7	0.434	0.0015	0.129	0.142	1.287
1.7×10^5	S.D.	1.6	0.037	0.0003	0.012	0.015	0.133
	N	5	5	5	5	5	5

Group / Dose (cells/animal)		Spleen	Kidney	Adrenal gland	Testis	Prostate
G1	Mean	0.064	0.331	0.0036	0.173	0.0069
0	S.D.	0.005	0.011	0.0004	0.028	0.0021
	N	5	5	5	5	5
G2	Mean	0.059	0.351	0.0047	0.191	0.0089
Saline: 0	S.D.	0.004	0.037	0.0010	0.023	0.0029
	N	5	5	5	5	5
G4	Mean	0.078 #	0.355	0.0038	0.176	0.0080
1.7×10^5	S.D.	0.013	0.049	0.0006	0.021	0.0025
	N	5	5	5	5	5

Significantly different from control by Steel test: # $p<0.05$.

Table S8-2. (Continued)

Sex: Female (g)

Group / Dose (cells/animal)		B.W.	Brain	Pituitary	Heart	Lung	Liver
G1	Mean	22.3	0.449	0.0025	0.112	0.143	1.152
0	S.D.	0.6	0.016	0.0004	0.007	0.008	0.108
	N	5	5	5	5	5	5
G2	Mean	22.3	0.461	0.0018	0.112	0.161	1.126
Saline: 0	S.D.	0.5	0.007	0.0005	0.006	0.016	0.065
	N	5	5	5	5	5	5
G4	Mean	23.4 *	0.453	0.0018 *	0.113	0.145	1.131
1.7×10^5	S.D.	0.7	0.016	0.0004	0.008	0.012	0.041
	N	5	5	5	5	5	5

Group / Dose (cells/animal)		Spleen	Kidney	Adrenal gland	Ovary	Uterus
G1	Mean	0.080	0.272	0.0074	0.0085	0.097
0	S.D.	0.009	0.021	0.0015	0.0009	0.030
	N	5	5	5	5	5
G2	Mean	0.079	0.275	0.0074	0.0086	0.090
Saline: 0	S.D.	0.006	0.009	0.0015	0.0006	0.030
	N	5	5	5	5	5
G4	Mean	0.079	0.263	0.0083	0.0078	0.089
1.7×10^5	S.D.	0.003	0.006	0.0021	0.0012	0.023
	N	5	5	5	5	5

Significantly different from control by Dunnett's t-test: * $p<0.05$.

Table S9-1. Mean Relative Organ Weights (Main group)

Sex: Male (g/100 g body weight)

Group / Dose (cells/animal)		B.W. (g)	Brain	Pituitary	Heart	Lung	Liver
G1	Mean	24.3	1.750	0.0061	0.516	0.568	5.213
0	S.D.	1.2	0.102	0.0011	0.031	0.042	0.200
	N	10	10	10	10	10	10
G2	Mean	24.5	1.753	0.0065	0.519	0.574	5.134
Saline: 0	S.D.	1.2	0.048	0.0020	0.036	0.045	0.669
	N	10	10	10	10	10	10
G3	Mean	24.6	1.732	0.0060	0.500	0.615	5.510
3.4×10^4	S.D.	1.4	0.086	0.0007	0.042	0.094	0.324
	N	10	10	10	10	10	10
G4	Mean	24.8	1.730	0.0065	0.527	0.577	5.322
1.7×10^5	S.D.	1.8	0.117	0.0012	0.037	0.049	0.410
	N	10	10	10	10	10	10

Group / Dose (cells/animal)		Spleen	Kidney	Adrenal gland	Testis	Prostate
G1	Mean	0.236	1.302	0.0168	0.723	0.0361
0	S.D.	0.020	0.052	0.0032	0.087	0.0188
	N	10	10	10	10	10
G2	Mean	0.260	1.353	0.0169	0.775	0.0336
Saline: 0	S.D.	0.051	0.101	0.0051	0.130	0.0148
	N	10	10	10	10	10
G3	Mean	0.262	1.296	0.0159	0.671	0.0401
3.4×10^4	S.D.	0.031	0.077	0.0040	0.069	0.0102
	N	10	10	10	10	10
G4	Mean	0.262	1.350	0.0181	0.757	0.0352
1.7×10^5	S.D.	0.036	0.094	0.0049	0.078	0.0123
	N	10	10	10	10	10

Table S9-1. (Continued)

Sex: Female (g/100 g body weight)

Group / Dose (cells/animal)		B.W. (g)	Brain	Pituitary	Heart	Lung	Liver
G1	Mean	22.2	2.044	0.0092	0.513	0.699	5.258
0	S.D.	0.9	0.107	0.0013	0.038	0.060	0.366
	N	10	10	10	10	10	10
G2	Mean	22.9	2.001	0.0089	0.511	0.690	5.227
Saline: 0	S.D.	1.3	0.077	0.0013	0.034	0.048	0.521
	N	10	10	10	10	10	10
G3	Mean	22.3	2.047	0.0099	0.527	0.698	5.106
3.4×10^{4}	S.D.	1.0	0.106	0.0033	0.055	0.029	0.483
	N	10	10	10	10	10	10
G4	Mean	22.2	2.062	0.0082	0.517	0.717	5.274
1.7×10^{5}	S.D.	1.1	0.097	0.0016	0.030	0.081	0.231
	N	10	10	10	10	10	10

Group / Dose (cells/animal)		Spleen	Kidney	Adrenal gland	Ovary	Uterus
G1	Mean	0.391	1.237	0.0370	0.0343	0.522
0	S.D.	0.033	0.098	0.0056	0.0056	0.170
	N	10	10	10	10	10
G2	Mean	0.369	1.190	0.0296	0.0270	0.463
Saline: 0	S.D.	0.041	0.065	0.0070	0.0039	0.189
	N	10	10	10	10	10
G3	Mean	0.424	1.207	0.0317	0.0314	0.515
3.4×10^{4}	S.D.	0.050	0.060	0.0064	0.0068	0.230
	N	10	10	10	10	10
G4	Mean	0.397	1.192	0.0333	0.0300	0.495
1.7×10^{5}	S.D.	0.054	0.094	0.0074	0.0099	0.159
	N	10	10	10	10	10

Table S9-2. Mean Relative Organ Weights (Recovery group)

Sex: Male (g/100 g body weight)

Group / Dose (cells/animal)		B.W. (g)	Brain	Pituitary	Heart	Lung	Liver
G1	Mean	25.9	1.634	0.0054	0.482	0.533	5.126
0	S.D.	1.3	0.087	0.0010	0.023	0.026	0.212
	N	5	5	5	5	5	5
G2	Mean	27.0	1.560	0.0051	0.477	0.535	5.194
Saline: 0	S.D.	1.3	0.071	0.0009	0.025	0.040	0.092
	N	5	5	5	5	5	5
G4	Mean	25.7	1.687	0.0058	0.501	0.551	5.013
1.7×10^5	S.D.	1.6	0.129	0.0010	0.037	0.030	0.551
	N	5	5	5	5	5	5

Group / Dose (cells/animal)		Spleen	Kidney	Adrenal gland	Testis	Prostate
G1	Mean	0.248	1.278	0.0139	0.667	0.0266
0	S.D.	0.010	0.045	0.0019	0.117	0.0086
	N	5	5	5	5	5
G2	Mean	0.218 #	1.296	0.0174	0.705	0.0331
Saline: 0	S.D.	0.004	0.098	0.0037	0.075	0.0115
	N	5	5	5	5	5
G4	Mean	0.303 #	1.374	0.0149	0.688	0.0309
1.7×10^5	S.D.	0.032	0.111	0.0030	0.101	0.0089
	N	5	5	5	5	5

Significantly different from control by Steel test: # $p<0.05$.

Table S9-2. (Continued)

Sex: Female (g/100 g body weight)

Group / Dose (cells/animal)		B.W. (g)	Brain	Pituitary	Heart	Lung	Liver
G1	Mean	22.3	2.014	0.0111	0.502	0.641	5.166
0	S.D.	0.6	0.065	0.0015	0.021	0.021	0.369
	N	5	5	5	5	5	5
G2	Mean	22.3	2.069	0.0080 *	0.504	0.725	5.054
Saline: 0	S.D.	0.5	0.070	0.0022	0.024	0.088	0.271
	N	5	5	5	5	5	5
G4	Mean	23.4	1.939	0.0076 *	0.484	0.621	4.847
$1.7×10^5$	S.D.	0.7	0.095	0.0020	0.047	0.059	0.272
	N	5	5	5	5	5	5

Group / Dose (cells/animal)		Spleen	Kidney	Adrenal gland	Ovary	Uterus
G1	Mean	0.359	1.219	0.0332	0.0381	0.436
0	S.D.	0.035	0.067	0.0073	0.0041	0.126
	N	5	5	5	5	5
G2	Mean	0.354	1.233	0.0334	0.0386	0.400
Saline: 0	S.D.	0.022	0.058	0.0064	0.0022	0.128
	N	5	5	5	5	5
G4	Mean	0.338	1.127 *	0.0357	0.0332	0.384
$1.7×10^5$	S.D.	0.018	0.031	0.0091	0.0049	0.108
	N	5	5	5	5	5

Significantly different from control by Dunnett's t-test: * p<0.05.

A.

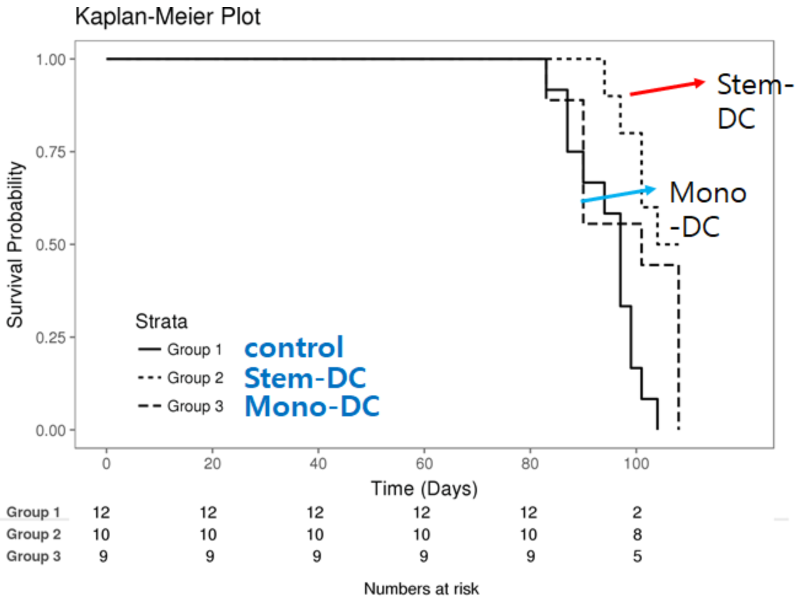

B

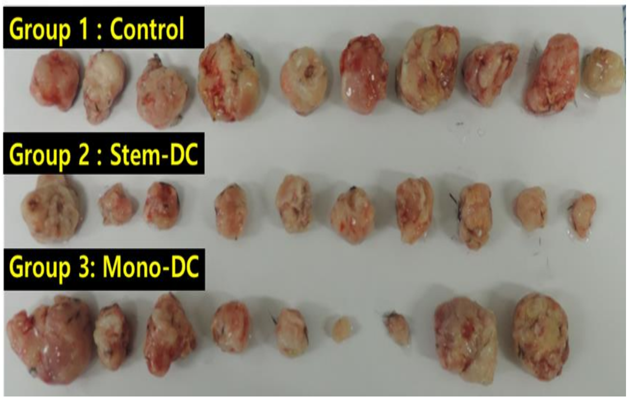

C

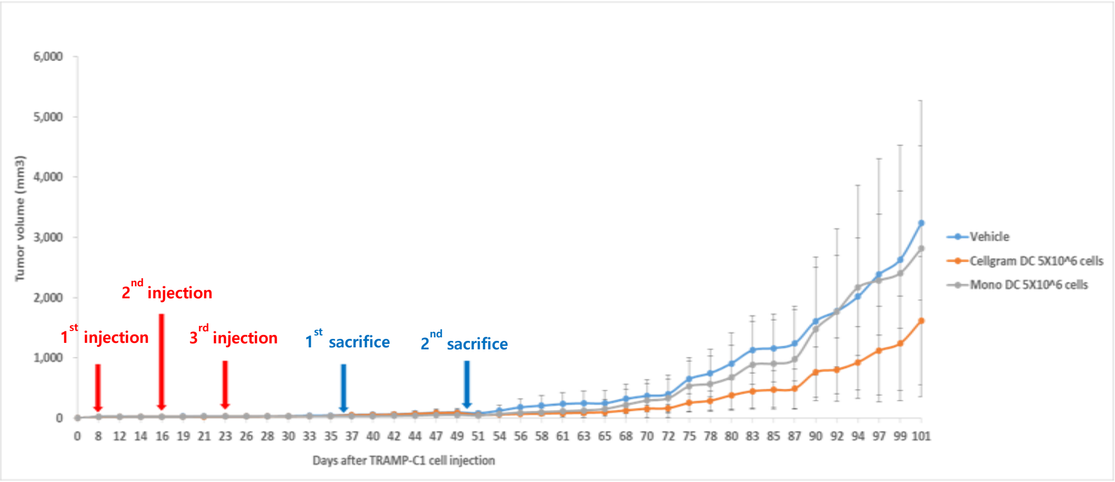

2.5. Confirmation of the safety of CellgramDC vaccine

Supplement 1. A 6-week repeated (1time/week) subcutaneous dose toxicity study with 2-week recovery period of CDW in C57BL/6 mice

Feed intake, ophthalmological examination, hematological examination, blood biochemical examination, organ weight, autopsy, and local tolerance test

Supplement 2. **A**: Survival probability, **B, C**: Tumor size and volume
